# Living standard and access to tetanus toxoid immunization among women in Bangladesh

**DOI:** 10.1186/s12889-022-13448-7

**Published:** 2022-05-24

**Authors:** Ummay Nayeema Islam, Kanchan Kumar Sen, Wasimul Bari

**Affiliations:** Department of Statistics, University of Dhaka, Dhaka, 1000 Bangladesh

**Keywords:** Tetanus, Tetanus toxoid, Living standard, Alkire andFoster methodology, MICS, Bangladesh

## Abstract

**Background:**

Although Bangladesh has an impressive track record in the reduction of maternal and child mortality, tetanus, a dreadful disease, impedes the way to achieve Sustainable Development Goal (SDG) in this respect. Sufficient doses of tetanus toxoid containing vaccine during pregnancy ensure immunity against tetanus to mothers as well as newborns. Since inequalities persist across vaccination programs globally, in this paper, an attempt has been made to examine whether tetanus toxoid immunization (TTI) status among the women of reproductive age in Bangladesh for their most recent live birth born preceding 2 years of the survey changes with their living standard index (LSI).

**Methods:**

Five domains of deprivation such as energy use, improved sanitation, drinking water, housing and assets ownership were used to compute the LSI using a approach proposed by Alkire and Foster. The adjusted association between LSI and TTI was established by using logistic regression model. For the purpose of statistical analysis, a nationally representative cross-sectional data extracted from Bangladesh Multiple Indicator Cluster Survey (BMICS), 2019 have been used.

**Result:**

The bivariate analysis revealed that 79.5% (95% CI 78.0–81.0) of women with low and 83.1% (95% CI 81.3–84.9) with moderate living standards had sufficient vaccination coverage for their most recent pregnancies while this percentage was higher for the women who belonged to high living standard (85.2, 95% CI = 84.2–86.2). A strong evidence for greater odds of sufficient immunization with TT among the women maintaining a high standard of living (AOR = 1.24, 95% CI = 1.08–1.42, *p* < 0.01) was found from regression analysis.

**Conclusion:**

The results depict existing living standard disparity with respect to TT vaccination coverage among women in Bangladesh. Present research suggests that immunization campaigns need to be conducted especially for the disadvantaged people to improve their health care and immunization service utilization among women within the age bracket of 15 to 49. This study proposed a scientific way to enhance TT vaccination among Bangladeshi women, which could help Bangladesh attain a widespread tetanus protection and thus, meet the SDGs for maternal and child mortality reduction.

## Background

In recent decades, the world has observed a remarkable progress in reducing newborns and maternal deaths. From 2000 to 2017, the global maternal mortality rate fell by approximately 38%, whereas the infant mortality rate dropped by nearly half, from 37 to 18 deaths per 1000 live births between 1990 and 2020 [1]. However, a large number of mothers and their newly born babies are still dying from various infectious diseases which are either preventable or treatable [1]. Tetanus is one of these infectious diseases which is also called lockjaw, is a serious nervous system infection caused by a spore-forming, anaerobic bacillus *Clostridium tetani* [2]. Definitions can vary as well based on when a child gets infected by tetanus. If a newly born child gets infected with tetanus within the first 28 days after birth, is called neonatal tetanus (NT) and if it occurs during pregnancy or within 6 weeks after pregnancy, it is maternal tetanus (MT) [3]. Maternal and neonatal tetanus (MNT), a major public health issue, affects women and their babies when women give birth to unsanitary conditions and do not receive a complete series of tetanus toxoid (TT) vaccines [2]. It was estimated that over 55,000 individuals died from this dreadful disease in 2015 alone, with the vast majority occurring in low- and middle-income countries [4]. Again in 2018, 25,000 neonates perished as a result of tetanus [5]. Though Government of Bangladesh has achieved MNT elimination status in June, 2008, tetanus still remains a significant health concern throughout the country [6]. This is because, according to WHO, tetanus cannot be eradicated as tetanus spores are present naturally in the environment, but the risk of being infected by it can be eliminated by immunizing neonates and women of reproductive age [7]. Moreover, underreporting of cases has made the situation worse and NT has been declared as one of the most under-reported infectious diseases [8]. A cross-sectional study of 149 (irrespective of age and gender) patients diagnosed with tetanus was conducted in Infectious Disease Hospital, Dhaka- which gave us a glimpse of present status of tetanus in the country. The result depicted an 8.7% (13 out of 149) of NT with a high fatality rate of 53.84% (7 out of 13) and patients were the least immunized with TT compared to those who survived [9]. Actual extent of death toll caused by tetanus is always undetectable in developing countries because in most cases newborns and mothers die at home and such incidents, either the birth or the death, are seldom reported to the proper authority [10].

WHO defines MNT elimination as less than 1 NT case per 1000 live births (LB) in each district and any district not meeting the criteria particularly for more than 1 year does not get the recognition [7]. To achieve this goal, Bangladesh has been working on Expanded Program on Immunization (EPI) under the technical support of WHO and UNICEF since April 7, 1979 [11]. EPI was initiated to monitor immunization services and guide strategies, in particular, for the eradication of six vaccine preventable diseases including tetanus [12]. In 1993, the government of Bangladesh approved the TT5 dose schedule for women of childbearing age, initially from 15 to 45 years of age, and then from 15 to 49 years of age [11]. Eligible women are identified through clinic-based and outreach initiatives as part of the EPI implementation, and health workers are largely responsible for administering immunizations at health centers [13]. Pregnant women and women of child-bearing age are the target demographic, who can protect themselves against MNT throughout their reproductive ages by a complete vaccination program of TT which will ensure protection of their newborns by transferring tetanus antibodies to the fetuses [5]. A systematic review of Blencowe et al. concluded that TT vaccine had an efficiency rate of 94% to prevent deaths from neonatal tetanus (NT) [14]. To decrease MNT deaths in particular, a global MNT elimination program was launched by UNICEF, UNFA and WHO in 1999 and declared TT vaccine as a safe public health intervention [15].

**Fig. 1:**
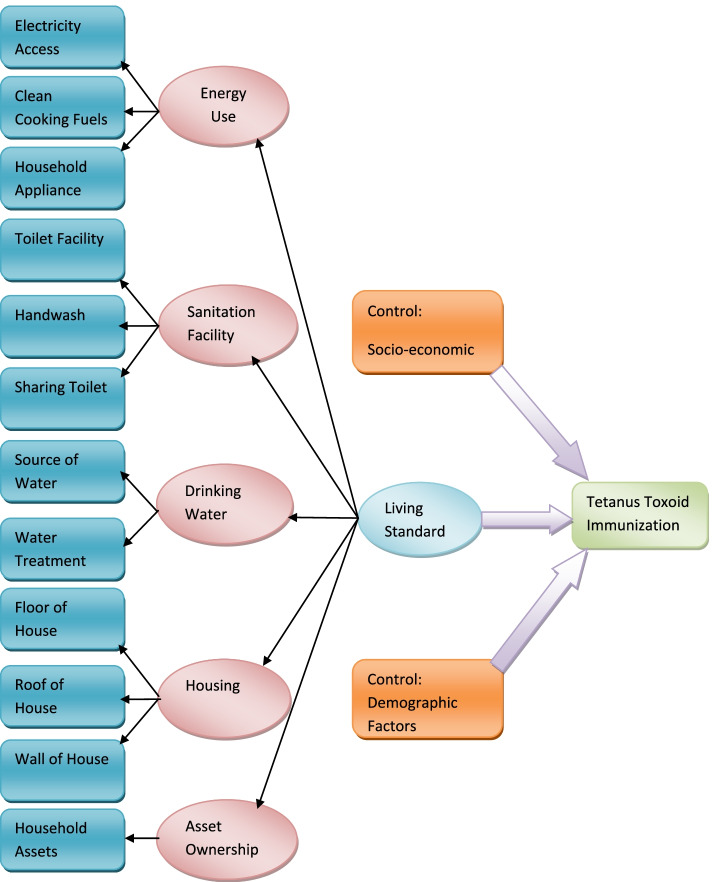
Conceptual framework of empirical association between living standard and tetanus toxoid immunization

In Bangladesh, inequalities in the access to vaccination services persist in terms of poverty and marginalization. As a consequence, health condition varies among different subgroups of the population. For instance, mortality rates of under-five range from 36 in the richest quintile to 55 in the poorest quintile [16]. By reviewing the inequalities in the health sector in Bangladesh, one study stated that although the country made significant improvement in reducing fertility, maternal and child mortality and malnutrition in the period of 2000s, the gap between the poor and the non-poor in respect to health indicators remained significant and unacceptably high [17] and the findings of the study echo from studies in Gabon and Afghanistan. A cross-sectional study in Gabon concluded that substantial gap existed between well-off and disadvantaged regarding maternal health-care services utilization [18]. Another study conducted in Afghanistan revealed that households belonging to richest groups were more exposed to utilization of antenatal health-care facilities [19]. Being inspired by the above evidences, this paper hypothesized that whether standard of living index was associated with tetanus toxoid-containing vaccine coverage among women of Bangladesh. The living standard of a woman was measured based on how much a woman was deprived under five indicators, namely, energy, drinking water, sanitation, housing and assets. The conceptual framework given in Fig. 1 depicted the causal pathway between exposure and outcome variable. This paper will guide policy makers to imply mass vaccination program of TT, consequently will help Bangladesh move forward and gain Sustainable Development Goal (SDG) 3.1 (reducing maternal mortality ratio to < 70 per 1000 LB) and SDG 3.2 (reducing neonatal mortality to 12 per 1000 LB) by 2030.

## Data and methods

### Data source

**Fig. 2:**
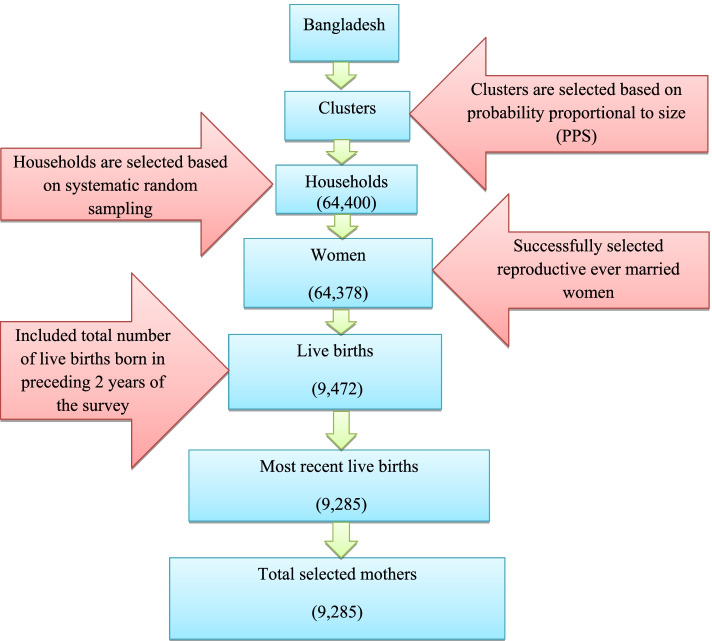
Flow chart of sample selection in the study

Data used in this study were extracted from individual women record of Bangladesh Multiple Indicator Cluster Survey (BMICS) 2019 database available at https://mics.unicef.org/surveys. The BMICS was first launched by the Bangladesh Bureau of Statistics (BBS) with the collaboration of United Nations Children’s Fund (UNICEF). Two stage stratified cluster sampling method was adopted to conduct the survey. The first stage evolved systematic selection of 3220 enumeration areas (primary sampling units) with probability proportional to size from rural and urban strata. Household listing and systematic selection of 20 households from each selected cluster were conducted at the second stage that comprised a total of 64,400 households. Of the households BMICS successfully interviewed 64,378 eligible women who were ever married and aged between 15 and 49 years. The scope of the interview was the complete history of these women’s live births including sex, month and year of each birth, survival status and age at the time of survey and age at death along with socio-economic and demographic variables. BMICS also concerned about maternal and child health related information. Since the focus of this manuscript was on the tetanus vaccination that they received for their most recent live births, the analysis of the study was restricted to 9285 women who had given birth within 2 years preceding the survey. The selection of sample was completely explained through flow chart in Fig. 2.

### Variables

#### Outcome variable

The outcome variable of interest for this study was Tetanus Toxoid Immunization (TTI) status, a binary random variable, having two categories-adequate and inadequate. This was assessed by asking a woman whether she had sufficient immunization with TT for her most recent live birth. A mother and her newborn were considered to be adequately protected against tetanus bacteria if a mother (i) received at least two doses of TT during the most recent pregnancy; or (ii) perceived two or more doses of which the last one was less than 3 years before the birth; or (iii) had at least three doses where the last dose was taken within 5 years prior to the particular birth or (iv) perceived four or more doses of TT with the last dose within 10 years before the delivery or (v) at least five doses at any time throughout the entire life period but prior to this recent birth [20].

#### Exposure variable

In this study, Living Standard Index (LSI) was considered as the primary exposure variable categorized into high, moderate and low living standard. The study used the technique of multidimensional poverty measures developed by Alkire and Foster to estimate LSI [21]. The indexing of well-being reflected the percentages of households which were deprived in the weighted deprivation scores. In terms of data availability, a household’s deprivation score was captured using five domains- energy, drinking water, sanitation, housing and household assets with relative weights assigned to each indicator. These five domains along with weights used in computing living standard index were given in Table 1. Mathematically, the deprivation score can be derived as follows

$${Y}_i=\left(\frac{1}{m}\sum_{j=1}^m\frac{1}{p_j}\sum_{k=1}^{p_j}{z}_{ijk}\right);i=1,2,\dots, n;j=1,2,\dots, m;k=1,2,\dots, {p}_j,$$

where *Y*_*i*_ is the score of *i*^*th*^ household, *m* is the number of dimensions, *p*_*j*_ is the number of indicators in *j*^*th*^ dimension, *z*_*ijk*_ is the binary value (1/0) of *k*^*th*^ indicator in *j*^*th*^ domain for *i*^*th*^ household, and *n* is the total number of households. Note that women coming from same households have same deprivation scores. The deprivation score in living standard lies between 0 and 1, where the higher value of score indicates low standard of living and lower value indicates high living standard. A threshold value (deprivation cut-off point) was predetermined to assess the category in which a household would fall. If the deprivation score was between 0.33 and 0.5, a household was considered to be moderate living standard; and a household is referred to as low living standard if the score is higher than 0.5. The household which does not exceed the cut-off point 0.33 is termed as high living standard.

**Table 1:** The dimensions, indicators, deprivation cutoffs and weights of household living standard

Dimension	Indicator	Deprivation	Weight
**Energy Use**	Electricity Access	The household has no access to electricity.	1/15
	Modern Cooking Fuels	The household has no clean cooking fuels (electricity, natural gas, kerosene or biogas).	1/15
	Household Appliance	The household does not own more than one of the following household appliances related to energy: water pump, air conditioner, electric fan, computer, mobile telephone, radio, TV and refrigerator	1/15
**Improved Sanitation**	Toilet Facility	The toilet facility of the household does not have the followings: flush toilet, flush to piped sewer system, flush to septic tank, flush to pit latrine, pit latrine with slab or ventilated improved pit latrine.	1/15
	Handwash	The household does not have the improved hand washing materials (liquid/bar soap or detergent)	1/15
	Shared Toilets	It shares toilet facilities with other households.	1/15
**Drinking Water**	Source of Water	The household does not have the improved source of drinking water (piped water, tube well/borehole, protected dug well, rainwater, tanker or bottled water).	1/10
	Water Treatment	It does not treat water to make safer for drinking.	1/10
**Housing**	Floor	The household does not have the following floor materials: parquet or polished wood, vinyl or asphalt strips, ceramic tiles or cement.	1/15
	Roof	The household does not have the following roof materials: metal, wood, calamine/ cement fiber, ceramic tiles, cement or roofing shingles.	1/15
	Wall	The household does not have the following wall materials: tin, cement, bricks, cement blocks or shingles.	1/15
**Assets Ownership**	Household Assets	The household does not own more than one of bus/car/truck/covered van, bike, almirah, sofa set, more than one sleeping room and lands for agriculture.	1/5
**Total**			1

#### Independent variables

Other covariates used in this paper were mother’s education (uneducated, educated), mother’s age at first birth (< 20, 20–34, > 34), parental age gap (< 5 years, 5–10 years, > 10 years), wanted last child (yes, no), place of residence (rural, urban), Attitude to Partner Violence Against Women (APVAW) (unaccepted, accepted), region (eastern, central, western), happiness index (unhappy, happy). Some variables were constructed from available information in the survey. Eight administrative divisions were grouped into three regions which would minimize regional error. Eastern Bangladesh comprises Chittagong and Sylhet divisions, western for Khulna, Rajshahi and Rangpur divisions and central for Dhaka, Barishal and Mymensingh. APVAW was measured as a composite variable consists of five items that were intended to assess the respondent’s acceptance of wife-beating. These items included (i) went outside without informing her husband (ii) neglected her children (iii) argued with her husband (iv) refused to have sex and (v) burned food. If a woman justified beating for any one of five reasons, APVAW was considered as accepted, otherwise unaccepted. BMICS 2019 included a question on subjective perception about happiness of life. Interviewees were given a card with five smiling faces on it with the categories “very happy,” “somewhat happy,” “neither happy nor unhappy,” “somewhat unhappy,” and “very unhappy”. This study created a happiness index as a dummy variable with two categories: happy (very and moderately happy responses) and unhappy (other three responses). The socio-economic and demographic variables mentioned above were selected based on their association with TT vaccination coverage in previous literature [18, 21–27].

### Statistical analysis

Data extraction, merging, variable recoding, and both descriptive and analytical analyses were performed using STATA version 14. Chi-square tests were carried out to examine the significant association of selected covariates and TTI status. Error bars were constructed to visually understand the differences in mean deprivation score across TTI status. Finally, binary logistic regression model was employed to observe both the unadjusted and adjusted effects of LSI as well as other independent variables.

## Results

### Descriptive analyses

**Fig. 3:**
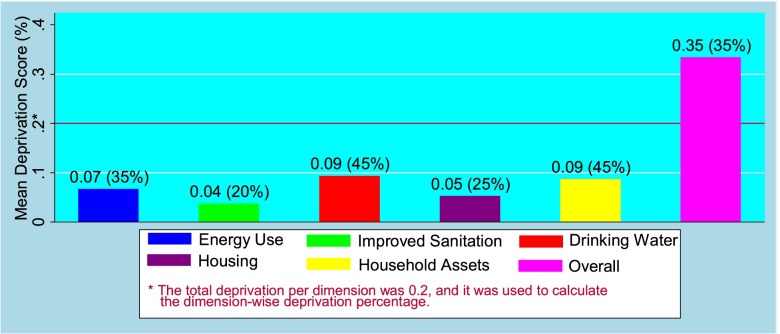
Average deprivation score along with deprivation percentage across the dimensions of living standard index

Bar diagram in Fig. 3 represented five bars portraying mean deprivation scores across five dimensions of main exposure variable (LSI). It was clear from this figure that overall mean deprivation score was 0.35. It implies that on the average, a household suffered from 35% deprivation in the dimensions. Among five domains, highest mean deprivation score (0.09) was observed in assets ownership and drinking water while least mean deprivation score (0.04) was found for improved sanitation. Moreover, the mean deprivation scores for energy use and housing were 0.07 and 0.05, respectively.

Table 2 depicted the sample characteristics and prevalence of adequate immunization with tetanus toxoid for each explanatory variable as percentages. Though it is expected that all mothers are immunized with TT, the prevalence of TTI in Bangladesh was found to be 83.2%. It was revealed that 28.9% of women belong to households which were multidimensional poor, whereas 52.9% women maintain high living standard. More than half of mothers (67.7%) had completed their secondary or higher level of education while only 32.3% of them had primary or no educational background. Most of the women (80.9%) lived in rural areas and 36.3% resided in the west region in Bangladesh. It was revealed that 62.1% of women had their first baby at the age of 35 or higher, whereas a few (8.6%) became mother of the first child at the age between 20 to 30 years. It was found that age difference between husband and wife was between 5 to 10 years for 51% women and more than 10 years for 22.1%. At the time of last pregnancy, 75.6% mothers wanted to be conceived. In terms of happiness index, 87.9% of mothers were satisfied with their current situation. When assessing women’s attitudes towards PVAW, 25.6% of women justified the wife-beating.

**Table 2:** Descriptive statistics of selected covariates and the distribution of Tetanus Toxoid Immunization (TTI) by the selected covariates along with *p*-value, BMICS 2019

Variables	Frequency n (%)	Prevalence of TTI	***P***-value
**Living Standard Index**			
Low	2681 (28.9)	79.5 [78.0–81.0]	
Moderate	1689 (18.2)	83.1 [81.3–84.9]	< 0.001
High	4915 (52.9)	85.2 [84.2–86.2]	
**Mother’s Education**			
Uneducated	2997 (32.3)	76.8 [75.3–78.3]	< 0.001
Educated	6288 (67.7)	86.2 [85.3–87.0]	
**Mother’s age at first birth (years)**			
< 20	2723 (29.3)	86.6 [85.3–87.9]	< 0.001
20–34	797 (8.6)	89.3 [87.2–91.5]	
35+	5765 (62.1)	80.7 [79.7–81.7]	
**Parental age gap**			
Within 5 years	2491 (26.8)	84.1 [82.7–85.5]	0.319
5–10 years	4738 (51.0)	82.9 [81.8–84.0]	
> 10 years	2056 (22.1)	82.6 [81.0–84.2]	
**Wanted last child**			
No	2267 (24.4)	80.0 [78.4–81.7]	< 0.001
Yes	7018 (75.6)	84.2 [83.3–85.0]	
**Place of residence**			
Urban	1774 (19.1)	82.9 [81.2–84.7]	0.768
Rural	7511 (80.9)	83.2 [82.4–84.1]	
**Intimate Partner Violence**			
No	6907 (74.4)	84.1 [83.2–85.0]	< 0.001
Yes	2378 (25.6)	80.4 [78.9–82.0]	
**Region**			
Eastern	2731 (29.4)	78.7 [77.2–80.2]	< 0.001
Central	3188 (34.3)	82.2 [80.9–83.5]	
Western	3366 (36.3)	87.7 [86.6–88.8]	
**Happiness index**			
Unhappy	1128 (12.2)	76.4 [73.9–78.9]	< 0.001
Happy	8157 (87.8)	84.1 [83.3–84.9]	
**Total**	**9285**	**83.2 [82.4–83.9]**	

**Fig. 4:**
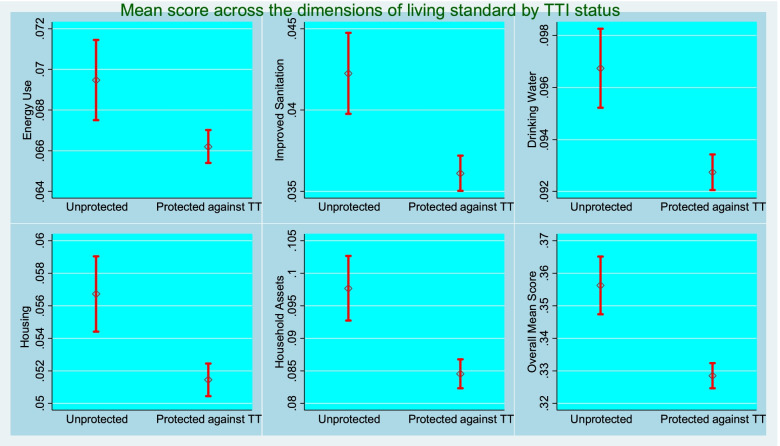
Multidimensional average deprivation scores by the tetanus toxoid status

Figure 4 showed point estimates along with error bars for five poverty measures across TTI status. It was observed that mean deprivation score was significantly higher for the women who had insufficient TT vaccine coverage compared to the group having sufficient TT vaccine coverage.

It was found that all the variables except parental age gap (*p*-value = 0.319) and place of residence (*p*-value = 0.768) had significant effect on having TT immunization.

It was observed that prevalence of TTI increases significantly (*p*-value< 0.001) as living standard of participant gets higher. Among the mothers with high living standard, 85.2% were immunized with TT, whereas it was 83.1 and 79.5% among the mothers with moderate and low living standard, respectively. Evidently, the rate of immunization was higher among educated mothers than uneducated mothers (86.2% versus 76.8%). Mothers of age group 20–34 years (89.3%) were vaccinated than their younger (86.6%) and older (80.7%) counterparts. Mothers were more immunized when they were likely to have the index pregnancy (84.2% versus 80.0%). Mothers supporting intimate partner violence were less prone in taking TTI (80.4% versus 84.1%). The prevalence of TTI was found to be highest (87.7%) in western region and lowest in eastern region (78.7%). Mothers who thought themselves happy were more willing to have immunization than their counterparts (84.1% versus 76.4%).

### Regression analysis

The study examined both the unadjusted and adjusted effects of living standard index on tetanus toxoid Immunization status. Table 3 presents the odds ratios (OR) with 95% confidence intervals obtained from binary logistic regression model. Note that unadjusted OR of being immunized with TT increases as living standard index increases. The odds of being immunized were significantly 27% (*p*-value< 0.01) and 48% (*p*-value< 0.001) higher for the mothers belonging to the moderate and high standard families, respectively compared to the mothers maintaining low living standard. After adjusting the demographic and relevant factors, it was still found that odds of immunization was 24% significantly (*p*-value< 0.05) higher for the mothers of high living standard families than mothers of low living standard families; whereas it was 15% higher for mothers having moderate living standard (*p*-value< 0.10).

**Table 3:** Unadjusted odds ratio (LSI only) and adjusted odds ratios with 95% CIs of all selected covariates associated with Tetanus Toxoid Immunization (TTI), BMICS 2019

Variables	UOR [95% CI]	AOR [95% CI]
**Living Standard Index**		
Low	1.00	1.00
Moderate	1.27 [1.08–1.48]**	1.15 [0.98–1.36]^+^
High	1.48 [1.31–1.68]***	1.24 [1.08–1.42]**
**Mother’s Education**		
Uneducated		1.00
Educated		1.47 [1.30–1.67]***
**Mother’s Age at First Birth**		
< 20		1.00
20–34		1.39 [1.08–1.80]*
35+		0.75 [0.66–0.86]***
**Spousal Age Gap**		
Within 5 years		1.00
5–10 years		0.89 [0.78–1.01]^+^
> 10 years		0.84 [0.72–0.99]*
**Wanted Last Child**		
Yes		1.00
No		0.81 [0.72–0.92]**
**Place of residence**		
Urban		1.00
Rural		1.09 [0.95–1.26]
**Intimate Partner Violence**		
No		1.00
Yes		0.85 [0.75–0.97]*
**Region**		
Central	•	1.00
Eastern		0.81 [0.71–0.92]**
Western		1.54 [1.34–1.78]***
**Happiness index**		
Unhappy		1.00
Happy		1.31 [1.11–1.53]**
***Likelihood Test (LRT) Statistic***	39.45***	290.0***

***p < 0.001, **p < 0.01, *p < 0.05,^+^*p* < 0.1

The educated mothers had 47% (*p*-value< 0.001) higher odds of having vaccinated with TT compared to the uneducated mothers. Women who had their first pregnancies during age 20–34 years were 1.388 times (*p*-value< 0.05) as likely to receive adequate doses of TT as younger mothers (< 20 years). On the contrary, women conceiving first baby after 34 years of age had 25% (*p*-value< 0.001) lower odds of having protection against tetanus compared to the teenage mothers. Though parental age gap was found to be insignificant in bivariate section but after controlling the effects of other covariates it was found as significant factor. The odds of receiving TTI decreased if parental age gap increased [for 5–10 years, OR = 0.89, *p*-value< 0.120; for > 10 years, OR = 0.84, *p*-value< 0.05]. Sufficient TT immunization was lower among the mothers who did not want their most recent pregnancies compared to those who wanted to get pregnant (OR = 0.81, *p*-value< 0.001). The chances of receiving TT immunization did not vary place of residence. The study observed significant result on sufficient TTI in terms of attitude to PVAW. Odds of receiving TTI was 14.6% (*p*-value< 0.05) lower among the women who justified wife-beating as acceptable.

In comparison with central, receiving adequate TT doses was higher among western women (OR = 1.54, *p*-value< 0.001) while opposite scenario was observed among eastern women (OR = 0.81, *p*-value< 0.001). The mothers who felt delightful themselves with overall conditions had 33% higher odds (*p*-value< 0.001) of having adequate TTI than those of mothers who were not happy.

## Discussion

Though Bangladesh has accomplished immense success in immunization program, full coverage of tetanus toxoid immunization for mothers is yet to receive. This study revealed that among 9285 potentially eligible women aged between 15 and 49, 83.2% had adequate protection to fight against tetanus infection during their most recent pregnancies, resulting in live births, within last 2 years prior to the interview time. This percentage was somewhat slightly lower than the percentage (83.5%) reported in BMICS 2019 [27]. In comparison to other countries, this result was similar to that of a study conducted in Sierra Leone (82.1%) [28], higher than the TT vaccination status recorded by studies in Pakistan (69%) [29] and Kenya (61.4%) [22], but lower than those of Nepal (85.9%) [30] and India (89%) [31]. This variation in percentages might occur due to cultural and socio-economic factors, such as knowledge and attitudes towards immunization, lack of motivation, availability of vaccination centers, methodological challenges in measuring vaccination coverage and prevailing political issues [23].

The results obtained from this study suggested that standard of living plays a significant role in access to full TT vaccination among Bangladeshi women. A secondary analysis of Sierra Leone revealed that higher wealth quintile had 50.9% greater odds of receiving sufficient TTI compared to those with the poorest (*p*-value< 0.05) [28]. Another study was conducted among Indian women which showed that probability of perceiving TT vaccination increased with higher socio-economic status [24]. An existing literature in Pakistan showcased that disparities in the usage of TT injections was conspicuous in terms of economic status [25]. The current research examined lower odds of achieving full vaccination status among the households belonging to moderate and low living standard. A possible explanation might be that people living in poverty like to spend time on activities which generate income rather than taking actions for preventive health care services like immunization [32]. On the other hand, less affordability and access to less health information among these women compared to those of high living standards may explain this association [33]. A recent analysis carried out by UNICEF stated that the poor pregnant women might be discouraged from seeking medical attention due to high costs of antenatal care and delivery services which might endanger the lives of the mothers and their babies [34]. Poor households lack the capability to bear transportation cost, consequently, the accessibility to distant community clinics becomes a huge barrier to them [19]. Since a complete package of TT doses requires multiple visits to health care facilities for complete immunization, transportation expenses might be unbearable for poor women if vaccination centers are relatively far away [35].

The result of multiple logistic regression model explained that mother’s education played an important role in the uptake of TT vaccines and this is in line with other literatures [25, 26]. Ability to utilize health care inputs to maintain health is a greater responsibility that lie on the shoulders of the educated mothers where education enhances women’s decision-making power and thus, empower them to have control over their lives and take decisions about their own health as well as their children, which in turn increases overall prevalence of TTI. Our study showed that women who had their first pregnancies within age 20 and 34 years, were more likely to receive adequate doses of TT in comparison to those who had their first babies at younger age (< 20 years). A possible reason could be that being pregnant at comparatively matured age, these women might have encountered the knowledge and benefits of immunization and its benefits for themselves and for their children. On the contrary, compared to teenage mothers, the study provided a surprising result with lower odds of perceiving TT for the women who gave first birth at age 35 or later. The study methods did not permit the reasons behind this to be explored. This could be because this age category might not effectively participate in different health-related concerns.

After statistical adjustment, parental age gap was negatively associated with the use of TT immunization. This study observed that nearly all the women were younger than their husbands and two-thirds were younger by 5 or more. This might be related to the patrilineality found in Bangladesh. In patrilineal societies, age differences between partners are relatively higher and such unions are arranged frequently by senior members of a family than by couples’ own preferences [36]. In this culture, males have the authority to take any decisions regarding their conjugal lives. The limited decision making power of women in this setting impedes them to attend maternal health care services including TTI [37].

It was found that unwanted pregnancy brought lower odds of taking sufficient doses of TT. The women who do not want to get pregnant at that time have negative impact on their physical and mental health as well as their quality of life [38]. If a mother does not want to conceive or does not want others to know (which can be the case if pregnancy was the result of rape or incest), she may not seek antenatal care, consequently, may not eager to receive adequate doses of TT [39]. Our findings suggested that respondent’s perception of wife beating was a significant factor for sufficient reception of TT doses. A woman’s attitude to wife-beating is regarded as a proxy for her perception of social status [40]. A woman who considers such violence “justifiable” underestimates her status in a society and accepts the right of her husband to control the behavior even by means of violence. This lower sense of self-respect may act as an impediment to access health-care services even when ideal care is mandatory at the time of reproductive period [41].

The regression model revealed that adequacy on TTI was considerably anticipated by the regions where women resided. Women living in west region had higher odds of participating in TTI program while women in eastern part had lower odds of having sufficient TTI compared to those living in central part of Bangladesh. These regional differences regarding TTI might occur due to infrastructural development, availability of health care services and access to information about vaccination [28].

A statistically non-significant association was observed between antenatal TTI and place of residence (rural versus urban) which was consistent with other literatures [22, 42]. Another covariate, happiness index turned out to be positively associated with TTI status. Happiness and health go hand in hand. Woman’s satisfaction of her surroundings has a huge impact on her healthy life-style which may drive her to make conscious about her own health as well as her unborn child, thus, receive adequate protection against bacterial infection.

To the best of our knowledge, this study is the first which has examined the statistical relationship between living standard index and adequacy of tetanus toxoid immunization among the women in Bangladesh. Though the most recent nationally representative data of BMICS 2019 have been used in this study, the measurement of TTI status was reported based on face-to-face interview of women which might lead to recall bias as well as social desirability bias on the response they provided about maternal health-care facilities resulting into misclassification and measurement errors. Moreover, this study cannot establish causal inferences due to cross-sectional nature of itself [28].

## Conclusion

In fine, the positive association between LSI and TTI status proved the existing health inequalities in Bangladesh. Additionally, it is recommended that disadvantaged people especially women of child bearing age are in highly in need of immunization campaigns to improve health care and immunization service utilization. Moreover, to sustain the status of maternal and neonatal tetanus elimination (MNTE), the importance of routine immunization will have to be emphasized. Policy makers should make women’s education a top priority. Poor-performing areas should be identified, and appropriate steps should be implemented. Making vaccination sites more “user-friendly”, increasing client and provider awareness of the schedule and effectiveness of TTI, and raising the prevalence of client-retained TT immunization cards could all help to improve TT coverage. According to current findings, intimate partner abuse has prevented women from participating in the TT immunization program. The government should take necessary legal actions against the perpetrators in order to eliminate violence against women, which would improve maternity service utilization, lowering the risk of maternal mortality and assisting Bangladesh in reaching SDG.

## Data Availability

Dataset for this study is open and publicly available at the official UNICEF MICS website (https://mics.unicef.org/surveys).
